# Wheat genotypic variation in dynamic fluxes of WSC components in different stem segments under drought during grain filling

**DOI:** 10.3389/fpls.2015.00624

**Published:** 2015-08-11

**Authors:** Jingjuan Zhang, Wei Chen, Bernard Dell, Rudy Vergauwen, Xinmin Zhang, Jorge E. Mayer, Wim Van den Ende

**Affiliations:** ^1^Agricultural Sciences, School of Veterinary and Life Sciences, Murdoch UniversityMurdoch, WA, Australia; ^2^State Key Laboratory of Soil Erosion and Dryland Farming on Loess Plateau, Institute of Soil and Water Conservation, Chinese Academy of Sciences and Ministry of Water ResourcesYangling, Shaanxi, China; ^3^Lab of Molecular Plant Biology, Institute of Botany and Microbiology, KU LeuvenLeuven, Belgium; ^4^Grains Research and Development CorporationBarton, ACT, Australia

**Keywords:** 6-kestose, fructan exohydrolase (FEH), fructan remobilization, grain weight (GW), stem segments, stem water soluble carbohydrates (WSC)

## Abstract

In wheat, stem water soluble carbohydrates (WSC), composed mainly of fructans, are the major carbon sources for grain filling during periods of decreasing photosynthesis or under drought stress after anthesis. Here, in a field drought experiment, WSC levels and associated enzyme activities were followed in different stem segments (peduncle, penultimate internode, lower parts of stem, and sheath) during grain filling. The focus was on two double haploid (DH) lines, DH 307 and DH 338, derived from a Westonia/Kauz cross, two drought-tolerant wheat varieties that follow different drought adaptation strategies during grain filling. The results showed that in irrigated plants, in the period between 20 and 30 days after anthesis (DAA), 70–80% of WSC were fructans. Before and after this period, the fructan proportion varied from 10 to 60%, depending on the location along the stem. Under drought, the fructan proportion changed, depending on genotype, and developmental stages. After anthesis, stem fructans accumulation occurred mainly in the peduncle and penultimate internode until 14 DAA in both DH lines, with clear genotypic variation in subsequent fructan degradation under drought. In DH 307 a significant reduction of fructans with a concomitant increase in fructose levels occurred earlier in the lower parts of the stem and the sheath, as compared to DH 338 or other stem segments in both lines. This was associated with an earlier increase of grain weight and thousand grain weight in DH 307. Spatiotemporal analysis of fructan dynamics and enzymatic activities in fructan metabolism revealed that several types of FEHs are involved in fructan remobilization to the grain under drought.

## Introduction

In recent decades, droughts have affected the wheat production worldwide. Severe drought can reduce wheat grain yield by more than 50% in Australia (Ji et al., 2010). In the wheat belt of Western Australia (WA), water deficit intensifies around anthesis until about 4 weeks later, and severe drought usually occurs from a month after anthesis to maturity (Conocono, 2002; Zhang, 2008). Thus, terminal drought is a major problem for wheat production in WA and other wheat-producing regions in Australia, and it is also an increasing risk for wheat production in many parts of the world (Zhang et al., 2009). A lack of efficient screening approaches for adaptation to erratic and unpredictable climate stands in the way of more efficient breeding for drought tolerance (Ji et al., 2010).

A drought tolerant screening approach in crops is not only focused on survival under drought, but also on yield maximization (Fleury et al., 2010). A tolerant wheat variety should deliver grain weight under a water-deficit environment. There are two types of carbon source for grain filling in wheat: current photosynthate in green tissues (predominantly in the flag leaf), and storage carbohydrates, in stems, and leaf sheaths (Schnyder, 1993). Storage carbohydrates supply carbon skeletons during the dark period of the diurnal cycle and energy for transportation, and they become gradually more important during the later stages of grain filling (Schnyder, 1993). Under terminal drought, storage carbohydrates become the major source for grain filling, as photosynthesis ceases (Bidinger et al., 1977). The levels of stem water soluble carbohydrates (WSC) have been suggested as one potential selection criterion for drought tolerance in wheat (Volaire and Lelièvre, 1997; Wardlaw and Willenbrink, 2000; Foulkes et al., 2007). However, stem WSC levels vary, depending on growth stages, and conditions, and the remobilization of stem WSC clearly differs between genotypes (Evans and Wardlaw, 1996; Ehdaie et al., 2006b; Ruuska et al., 2006; Zhang et al., 2009, 2015). Therefore, the efficiency of stem WSC remobilization to grain needs to be further defined (UŽík and Žofajová, 2006; Rattey et al., 2009).

To better understand the remobilization of stem WSC to the developing grains, wheat stems were separated into different segments: peduncle, penultimate internode, and lower internodes. The time-dependent dry matter and WSC dynamics of each segment were studied before (Ehdaie et al., 2006a,b; Ma et al., 2014; Zhang et al., 2014). For both drought and irrigated plants, dry matter of the lower internodes contributed the most (55%), as compared with the penultimate internode (27%), and the peduncle (18%) (Ehdaie et al., 2006a). Under severe stress, the remobilization efficiency of dry matter increased by 137% in the lower internodes, compared to 33% in the upper internodes (Ma et al., 2014). At mid-grain filling, the WSC levels in the lower internodes were higher than those in the peduncles of both well-watered and drought stressed plants (Zhang et al., 2014). On average of drought and irrigated plants, the WSC in the lower internodes mobilized most of stem WSC (51%), followed by the penultimate internode and the peduncle (Ehdaie et al., 2006b). In the well-watered plants, the maximum WSC level in the penultimate internodes and the peduncle was reached 20 days after anthesis (DAA), while in the lower internodes it peaked at 10 DAA (Ehdaie et al., 2006b).

Stem WSC, composed mainly of fructans, are a long-term carbon storage form in the vegetative tissues of temperate grasses (Pollock, 1986; Pollock and Cairns, 1991). Stem fructans not only function as a carbon source for grain filling, but may also be used as part of recovery mechanisms under biotic and abiotic stresses (Schnyder, 1993; Yang and Zhang, 2006; Valluru and Van den Ende, 2008; Livingston et al., 2009). Small fructans were proposed as phloem-mobile signaling compounds under stress, contributing to stress tolerance and disease prevention (Van den Ende, 2013). Wheat graminan-type fructans contain both β-(2-1) and β-(2-6) linkages, with the latter being the predominant linkage in stems (Pollock and Cairns, 1991). Three types of fructan-synthesizing enzymes are involved in wheat fructan metabolism, namely 1-SST (sucrose: sucrose 1-fructosyltransferase), 1-FFT (fructan: fructan 1-fructosyltransferase), and 6-SFT (sucrose: fructan 6-fructosyltransferase). Linkage-specific FEHs (fructan exohydrolases 1-FEH, 6-FEH, and 6&1-FEH) degrade these fructans to sucrose and fructose (Van den Ende et al., 2003; Kawakami et al., 2005; Van Riet et al., 2006, 2008; Xue et al., 2008). Finally, soluble acid invertases (INVs) may be involved in hydrolysis of sucrose and yielding hexoses involved in osmoregulation or energy requiring processes (phloem loading, defense, and respiration) (Ruan, 2014).

Here, we studied the dynamic flux of the different stem WSC components to get a more detailed picture of their remobilization from different wheat stem segments to the grain. Compounds analyzed during grain filling, under drought and irrigated conditions, included sucrose, glucose, fructose, and different types of fructans. We also analyzed enzymes involved in fructan biosynthesis and degradation as well as INVs, with the objective of identifying the enzymes and genes mainly responsible for WSC remobilization to the grain. One goal is to find variants of these genes leading to increased WSC remobilization that could be used in marker-assisted breeding programs for drought tolerance. Specific patterns in the accumulation and degradation of fructans and by-products could also serve as phenotypic markers to be used when screening for drought adaptation.

## Materials and methods

### Plant materials

Doubled haploid lines DH 307 and DH 338 were selected from a population of 225 lines derived from a Westonia/Kauz cross. Westonia is a variety developed in Western Australia for consistent high yield in medium and low rainfall regions. Kauz was developed by the International Maize and Wheat Improvement Center (CIMMYT, Mexico) (Butler et al., 2005) for drought tolerance. Both varieties have high stem WSC levels (~40%) after anthesis. Kauz, however, senesces earlier at the similar water-deficit levels as compared with Westonia (Zhang et al., 2009). DH 307 and DH 338 were selected based on thousand grain weight (TGW) and kernel number (KN) differences. DH 307 resembles Westonia, with high TGW, while DH 338 is more like Kauz, with high KN per spike. Genetic diversity of the double haploid population was calculated based on 195 single-sequence repeat (SSR) markers and two *Rht-B1* and *Rht-D1* gene markers by non-metric multi-dimensional scaling (MDS). The results show that DH 307 is genetically close to Westonia while DH 338 is close to Kauz (Figure S1 in Zhang et al., 2015). Genetic dissimilarity based on MDS was calculated using the Numerical Taxonomy System (NTsys) v2.2 and Plymouth Routines in Multivariate Ecological Research (PRIMER v6).

### Field experiments

The field drought trial was carried out in 2011 at Merredin field station, Western Australia (31.5°S, 118.3°E). DH 307 and DH 338 were planted together with other DH lines in 5 m^2^ plots, in a randomized trial with three replicates sown on the 25th of May for both the drought and irrigated treatments (Zhang et al., 2015). The drought treatment was set up under rainout shelters, while the irrigated treatment was outside. Drought treatment was initiated at the average anthesis time (the 4th of September, 2011) of those DH lines. Besides 29 mm of rainfall, irrigated plots received 20 mm water on a weekly basis until three weeks after anthesis. Twelve neutron probes (down to 1.5 m depth) were distributed evenly in each treatment block to monitor soil moisture. In the drought treatment, soil water content was reduced by 30% at 10 cm depth at 15 and 20 days after anthesis (DAA) in DH 338 and DH 307, respectively (Zhang et al., 2015).

### Plant harvest

Four main stems of each plot were sampled weekly between 11:00 and 17:00 (Zhang et al., 2008). The harvest dates were the 30th of August, 6th, 14th, 20th, 28th of September, 4th and 14th of October in 2011. Since the anthesis dates of DH 307 and DH 338 were the 31st of August and 8th of September, DH 307 was sampled at -1, 6, 14, 20, 28, 34, and 44 DAA while for DH 338 was sampled at −9, −2, 6, 12, 20, 26, and 36 DAA (Table S1).

The samples were immediately placed on dry ice and subsequently stored in a −20°C freezer. Four frozen main stems per sample were sectioned into peduncle, penultimate internode, lower parts of the stem, and sheath. The four stem parts were then mixed together and cut into pieces about 5 mm long. The samples were then subdivided for storage at −80°C for enzyme analysis or freeze dried at −20°C and then oven-dried at 75°C for WSC and WSC components analysis.

### Carbohydrate analysis

On a weekly basis, total WSC were extracted from each segment sample (48 samples per week) using boiling deionized water and quantified by colorimetry using the anthrone reagent (Fales, 1951; Yemm and Willis, 1954). The WSC content was analyzed as previously described (Zhang et al., 2015). WSC components separated by high-performance anion exchange chromatography with pulsed amperometric detection (HPAEC-PAD) were quantified using the peak area with external standards for glucose, fructose, sucrose, 1-kestose, 6-kestose, neokestose, nystose, and bifurcose. Total fructan concentration was calculated as the WSC concentration (as determined by the anthrone method) minus the glucose, fructose, and sucrose. Total stem WSC concentration of selected samples was also determined by mild acid hydrolysis (Verspreet et al., 2012), which yielded the same results as the anthrone colorimetric method (data not shown). For reducing the work load, the week two samples were excluded as the study focused on the stage of stem WSC remobilization. In week 6, only one replicate for each treatment and DH line was analyzed.

### Enzyme activity measurements

Protein extraction and enzyme activity measurements were conducted as previously described (Zhang et al., 2015). For each DH line and under the two treatments, four segment samples in one replicate were analyzed.

### Statistical analysis

Phenotype data were analyzed by multivariate analysis of variance (MANOVA) using the general linear model implemented in PASW v 17 (California State University Information Services, Los Angeles, USA). Wilks Lambda was used as the multivariate test statistic. *Post-hoc* Tukey's Multiple Range tests were used to identify significant groupings. The Student's *t*-test was used in data pair significant analysis.

## Results

### Fructan reduction associated grain assimilation

**Figure 1:**
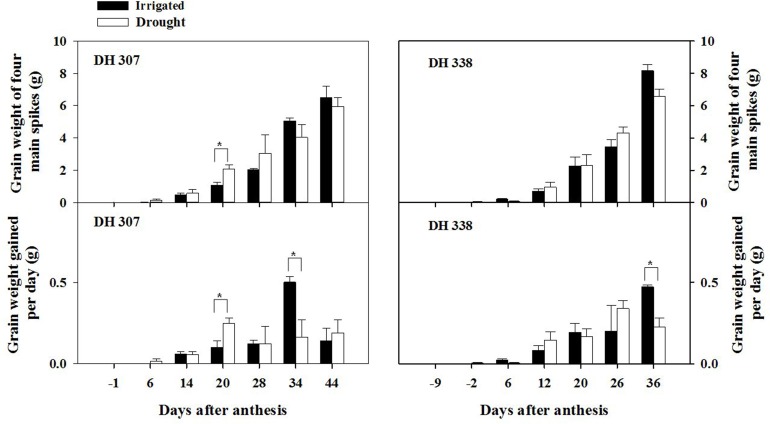
**Time-dependent grain weight and daily grain weight gains for DH 307 and DH 338 under irrigated and drought conditions in the field (DH 307: 6, 14, 20, 28, 34, and 44 DAA; DH 338: −2, 6, 12, 20, 26, and 36 DAA)**. The vertical bars represent SE. An asterisk (^*^) identifies significantly different values at *P* < 0.05.

**Figure 2:**
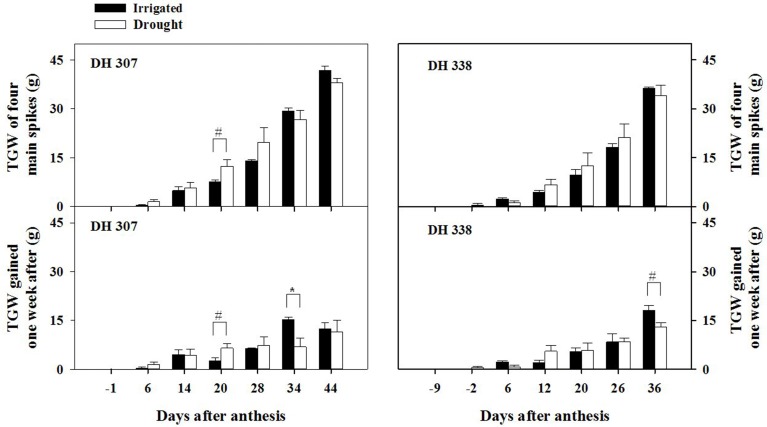
**Thousand grain weight (TGW) and daily increments of TGW in DH 307 and DH 338 under irrigated and drought conditions in the field (DH 307: 6, 14, 20, 28, 34, and 44 DAA; DH 338: −2, 6, 12, 20, 26, and 36 DAA)**. The vertical bars represent SE. An asterisk (^*^) identifies significantly different values at *P* < 0.05, and a pound sign (#) at *P* < 0.1.

Yield losses under drought (expressed as grain weight for four main spikes) were higher in DH 338 compared with DH 307 (Figure 1). GW of developing seeds at 20 DAA in DH 307 was significantly higher under the drought treatment than under irrigation. Accordingly, daily GW in drought stressed DH 307 was significantly higher (*p* < 0.05) at 20 DAA as compared to irrigated plants while no such difference was noted for DH 338. There was also a large increase (*p* < 0.1) in TGW at 20 DAA in DH 307 under the drought treatment while in DH 338 TGW was similar between treatments (Figure 2). In both DH lines, daily GW gains were significantly higher in irrigated plants between 30 and 40 DAA (*p* < 0.05) (Figure 1). A similar significant difference (*p* < 0.05) in daily TGW gains over the same period was recorded for DH 307, but no significant differences could be detected for DH 338 (Figure 2). In both DH lines KN per main spike increased until 20 DAA but remained steady afterwards under both water regimes (data not shown). No significant differences were detected, although the final KN of DH 338 (~50) tended to be higher than that of DH 307 (~40).

**Figure 3:**
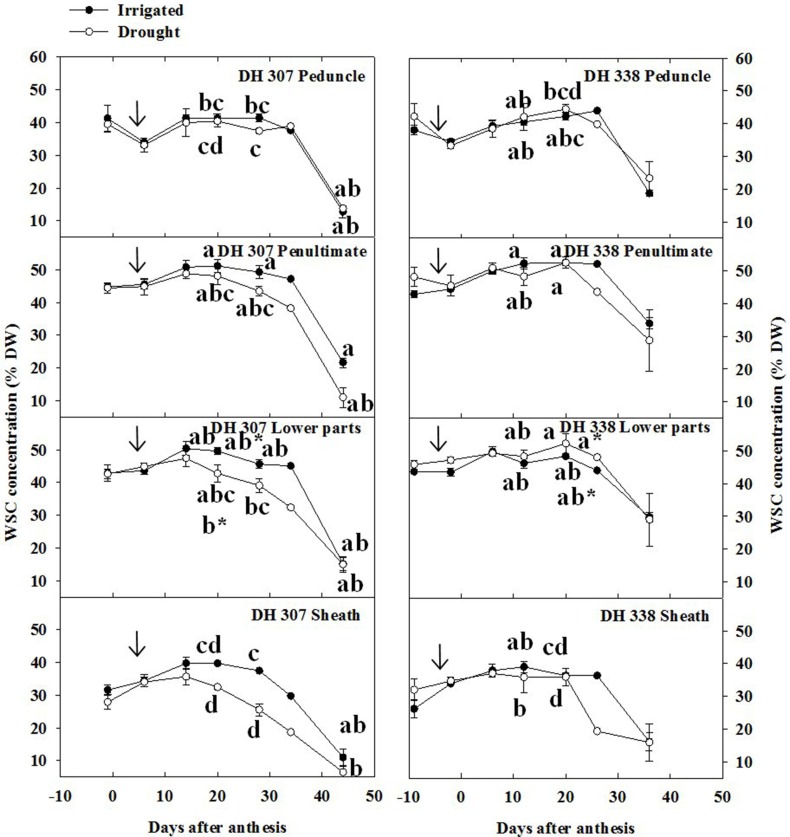
**Stem water soluble carbohydrate (WSC) levels in different stem segments of DH 307 and DH 338 under drought (open circles) and irrigated conditions (closed circles) in the field**. An asterisk (^*^) is a comparison at 20 days after anthesis between the two DH lines. The vertical bars represent SE. Values with the same letter are statistically not different at *P* = 0.05. Arrows indicate start of drought treatment.

Under drought, grain assimilation relies mainly on storage carbon present in the stem (Bidinger et al., 1977; Li et al., 2013), which prompted us to quantify WSC levels along the stem. Peak levels of WSC (ca. 50%) were slightly higher in the penultimate internode and lower parts of stem as compared to that in the peduncle (ca 45%), while the WSC level in the sheath was about 40% in both DH lines and under both treatments (Figure 3). Under drought, the WSC level of DH 307 decreased steadily in the penultimate internode, lower parts, and sheath after 15 DAA under drought, while a later (starting 20 DAA) and more prominent decrease was observed in DH 338. At 20 DAA, significantly lower WSC levels were found in the lower parts of drought treated DH 307 plants but not in DH 338 (Figure 3).

**Figure 4:**
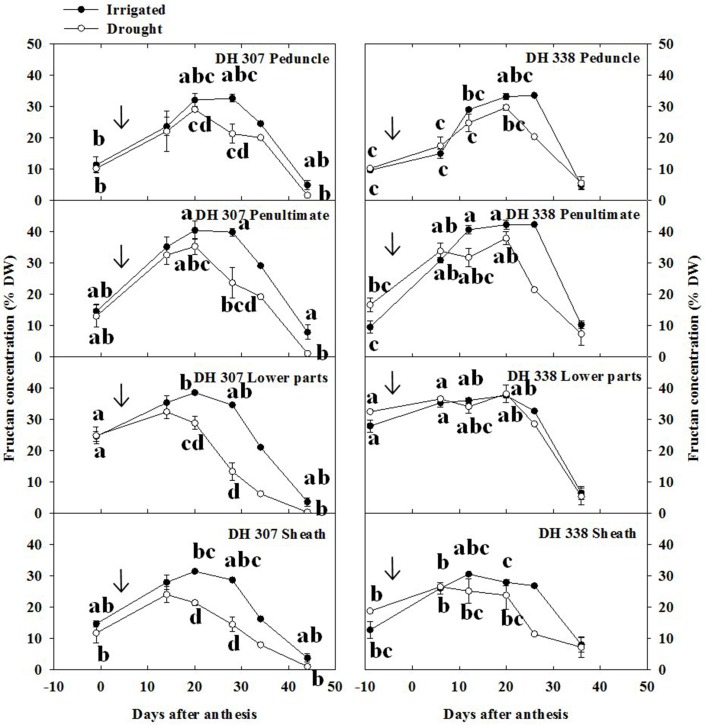
**Fructan concentration in different segments in DH 307 and DH 338 under drought (open circles) and irrigated conditions (closed circles) in the field**. The vertical bars represent SE. Values with the same letter are statistically not different at *P* = 0.05. Arrows indicate start of drought treatment.

Fructan levels represent a major class within wheat stem WSC (Pollock and Cairns, 1991) and were therefore quantified as part of the analysis. Peak levels of total fructans were higher in the penultimate internode and lower parts as compared to peduncle and sheath, especially in irrigated plants (Figure 4). The decreasing patterns in total fructan levels were similar but more pronounced as compared to the WSC levels (Figure 3) in both treatments and DH lines (Figure 4). Under drought, fructan degradation started earlier in DH 307 than in DH 338, especially in the lower parts and the sheath (Figure 4). The very sharp fructan decrease in the lower parts between 20 and 28 DAA in the drought treated DH 307 suggests that remobilization of this fructan pool could be of particular importance for grain filling.

**Figure 5:**
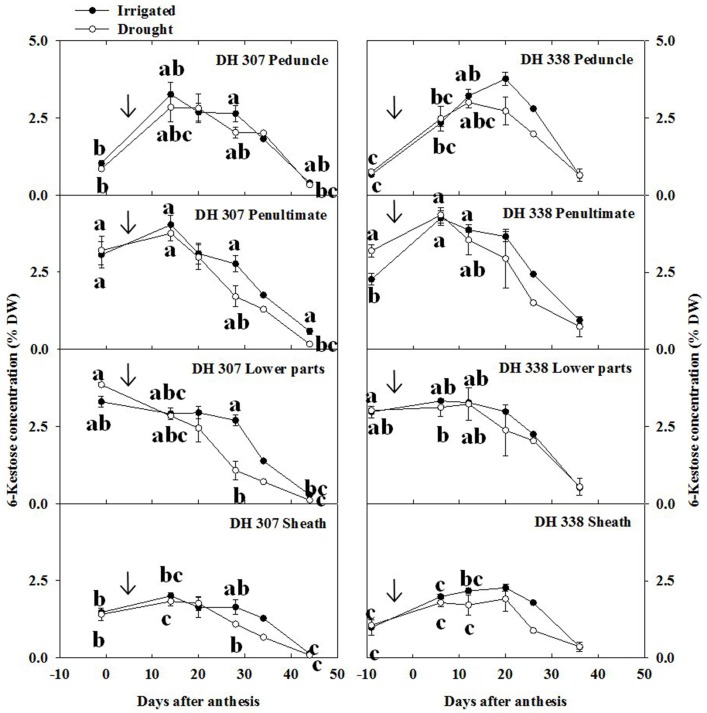
**6-Kestose concentration in different stem segments in DH 307 and DH 338 under drought (open circles) and irrigated conditions (closed circles) in the field**. The vertical bars represent SE. Values with the same letter are statistically not different at *P* = 0.05. Arrows indicate start of drought treatment.

In the peduncle, 6-kestose increased sharply up to 15 DAA and decreased gradually afterwards in DH 307 for both treatments, but in DH 338 such decrease was only observed under drought (Figure 5). In well-watered DH 338 the 6-kestose level rose until 20 DAA (Figure 5). In the penultimate internode, the level of 6-kestose increased slightly and dropped speedily afterwards in both DH lines and under both treatments. Under drought, the decrease of 6-kestose was significantly faster in the lower parts in DH 307 while in DH 338 there was no significant difference between treatments (Figure 5). Drought also led to a faster reduction in 6-kestose in the sheath in both DH lines (Figure 5).

Bifurcose levels decreased steadily in all stem segments from pre-anthesis to maturity in both DH lines and for both treatments (Figure S1). Before anthesis, levels were higher in the lower parts and penultimate internode (Figure S1). Levels of 1-kestose were below 1% in all stem segments and much lower than 6-kestose and bifurcose, decreasing slightly in both DH lines and under both treatments (data not shown).

### Fructose, glucose, and sucrose dynamics

**Figure 6:**
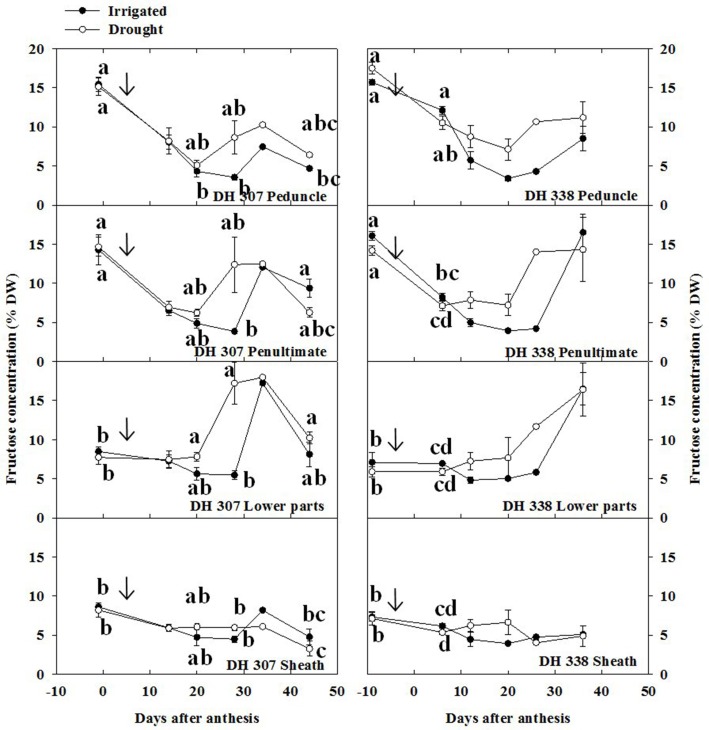
**Fructose concentration in different stem segments in DH 307 and DH 338 under drought (open circles) and irrigated conditions (closed circles) in the field**. The vertical bars represent SE. Values with the same letter are statistically not different at *P* = 0.05. Arrows indicate start of drought treatment.

Since sucrose is the main transport sugar in plants that can be easily converted to glucose and fructose by INV (Ruan, 2014), and FEH-mediated fructan degradation produces fructose and some sucrose, the levels of sucrose, fructose, and glucose were analyzed along with total fructan levels. Under drought, fructose levels increased in both DH lines after 20 DAA, especially in the peduncle, penultimate internode and lower parts (Figure 6). The highest fructose level in the lower parts of DH 307 (at 28 DAA) under drought was more than three times higher than in irrigated plants (Figure 6). Under well-watered conditions, fructose levels in both DH lines increased at later stage (after 30 DAA) in the lower parts, penultimate internode, and peduncle (Figure 6).

In both DH lines, glucose levels in drought-treated plants tended to be slightly higher than those in irrigated plants after 20 DAA (Figure S2), but these differences remained much smaller than for fructose (Figure 6). Overall, sucrose levels were below 5% and remained relatively stable in both lines and for both treatments (Figure S3), although under drought the sucrose level in the DH 307 peduncle was found to be significantly higher at the very end of the sampling period.

### Relative changes in WSC components

**Figure 7:**
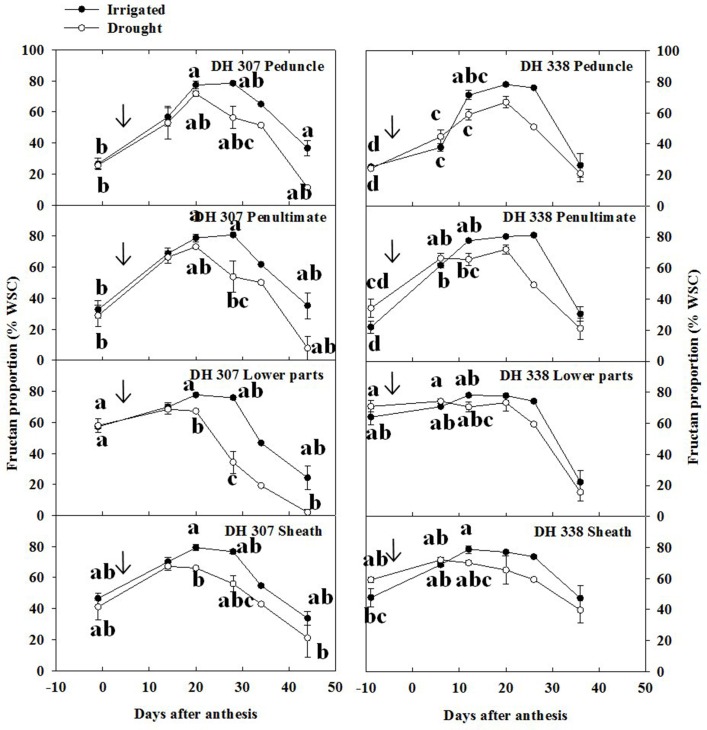
**Fructan proportion (% WSC) in different stem segments in DH 307 and DH 338 under drought (open circles) and irrigated conditions (closed circles) in the field**. The vertical bars represent SE. Values with the same letter are statistically not different at *P* = 0.05. Arrows indicate start of drought treatment.

The relative content of fructans within stem WSC varied as a function of time (Figure 7). Different from WSC levels (Figure 3), relative fructan levels generally increased before 20 DAA in both DH lines and under both treatments, especially in the peduncle and the penultimate internode (Figures 4, 7). Before anthesis, relative levels of hexoses and sucrose (WSC minus fructans) accounted for about 80 and 75% of stem WSC in the peduncle and penultimate internode, respectively (Figure 7). This proportion was much lower in the lower parts of the stem and the sheath, respectively (Figure 7). Thereafter, the absolute amounts of fructose and glucose decreased from 11–17% to 4–7% (Figure 6, Figure S2) while before 20 DAA the fructan proportion increased from 20–30% to 50–70% in the peduncle and penultimate internode, respectively (Figure 7). For both DH lines, the maximum relative fructan levels (70–80% of WSC) were reached between 20 and 30 DAA in irrigated plants. Before and after this period, the fructan proportion varied between 20 and 60% of WSC, depending on the stem segment (Figure 7). During fructan remobilization under drought (20–45 DAA), the fructan proportion decreased sharply, but earlier in DH 307 than in DH 338. Significant differences between drought and irrigated treatments were found in the lower parts and the sheath of DH 307 but not for DH 338 (Figure 7).

### Fructan and sucrose metabolic enzymes

In monocots, fructan dynamics are regulated by the balance between fructan biosynthetic and breakdown enzymes (Van den Ende et al., 2003). Therefore, the activities of the enzymes involved in these processes (expressed on a fresh weight basis) were investigated on the same samples that were used for WSC component analysis. Despite the presence of some segment-specific patterns, in general all the fructan biosynthetic enzyme activities (gradually) increased up to about 15–20 DAA, after which they (gradually) decreased again (Figures S4, S5). The drought treatment reduced the activity of these enzymes in a number of cases, but not reproducibly, and segment-specific variations were detected in both lines (Figures S4, S5).

Under drought, 1-FEH activity tended to increase in the peduncle and the penultimate internode after 14 DAA, while it remained similar between treatments in the lower parts and sheath in DH 307 (Figure S6A). 1-FEH activities were higher in all DH 338 stem segments under drought, as compared to irrigated plants after 10 DAA, although in the sheath the difference disappeared during the later stages (Figure S6A). Overall, 6-FEH patterns were rather similar as compared to those of 1-FEH (Figures S6A,B) although two differences also appeared (i) for DH 307, no differences were observed between both treatments and (ii) for DH 338, treatment differences were less clear in the 15–30 DAA period. Overall, the results indicate that the combined 1-FEH and 6-FEH activities are particularly important during the later stages in drought treated DH 338. INV activity in peduncle, penultimate internode, and lower parts of stem remained similar between treatments in both DH lines, but was lower compared to the sheath (Figure S7). In DH 307, INV activity was reduced in the sheath under drought relative to irrigated plants while it was similar in DH 338 under drought (Figure S7).

The absence of strong 6-FEH activity increases (on a fresh weight basis), especially in the lower parts (Figure S6B), during the most critical period of the onset of fructan degradation and fructose accumulation under drought (20–28 DAA; Figures 4, 6) was surprising. Therefore, we carefully considered 1-FEH and 6-FEH activity dynamics expressed on a total protein basis in this most critical time window, and compared them to the activities expressed on a fresh weight basis (Table S2). On a total protein basis, 1-FEH and 6-FEH activities in the lower stem of DH 307 under drought increased 3 and 4-fold, respectively, while this was not observed under irrigated conditions (Table S2). It should be noted that the increase of this specific activity relies on the lowered protein level in DH 307 after 28 days. Interestingly, for DH 338, under the drought treatment 1-FEH and 6-FEH activity increases were much less pronounced but increased strongly in the well-watered plants (Table S2). This suggests that within the critical period of 20–28 DAA, FEH dynamics under drought may play a more essential role in DH 307 than in DH 338, but this requires further investigation. The results in Table S2 should be taken with caution, since they are based on single sample analyses, although each sample represents a mixture of four stem segments originating from four different stems.

## Discussion

It is widely accepted that in wheat the stem fructan pool delivers carbon skeletons to the developing grain, and that this is of particular importance under terminal drought (Wardlaw and Willenbrink, 2000; Zhang et al., 2014). Wheat stems accumulate mainly branched, graminan, and levan-type fructans, which means that β-(2-6) linkages predominate (Bancal et al., 1993; Bonnett and Simpson, 1995; Van den Ende et al., 2003). Fructan accumulation in wheat stems after anthesis has been described before (Blacklow et al., 1984; Schnyder, 1993; Gebbing, 2003; Zhang et al., 2015), but at the same time the decrease in hexose levels in the peduncle and penultimate internode had not been described as yet.

As one glucose unit is produced for each fructan formed (Pollock, 1986), the glucose excess needs to be reconverted to sucrose (Edelman and Jefford, 1968). Hogan and Hendrix (1986) suggested that a substantial part of the glucose formed would be used for other mechanisms, such as for cell wall synthesis (Ordin and Hall, 1968; Hogan and Hendrix, 1986). It was noted that peduncle elongation proceeds up to 10 DAA (Gebbing, 2003; own results).

In general, fructan biosynthetic enzymes contributing to graminan accumulation increased during pre-anthesis and early post anthesis (up to about 20 DAA). Drought negatively affected these activities in DH 307 stem segments, while this was generally not the case in DH 338. This finding already pointed at genotype and stem-segment specific mechanisms under drought during this early period. However, the following period, 20–30 DAA, identified earlier as most critical for graminan remobilization (Zhang et al., 2015), revealed much more prominent drought-associated differences between the two DH lines under consideration.

Compared to irrigated plants, GW and TGW in DH 307 increased faster between 20 and 30 DAA. This correlates well with the significant decrease of fructan levels and spectacular increases of fructose in the lower parts of the stem and the sheath in DH 307, suggesting that these parts may play a more prominent role in delivering carbon to the grain.

It is generally believed that fructose is not phloem mobile. Since sucrose is the transport sugar (Ruan, 2014), FEH-mediated fructose production in the stem needs to be accompanied by sucrose synthesis (Willenbrink et al., 1998) prior to phloem loading (Lemoine et al., 2013) and delivery to the grains (Wardlaw and Willenbrink, 2000). Strikingly, no such drought-induced differences in fructan and FEH dynamics were observed in the lower parts of the stem in DH 338, suggesting that the two DH lines differ in remobilization efficiency over the time period of observation. However, it should be noted that FEH-mediated remobilization mechanisms are also of great importance in DH 338, but at a later stage. For instance, the sharp reduction of stem fructan levels between 25 and 40 DAA was also associated with GW gains after 30 DAA, especially for well-watered DH 338 plants.

Similar, 1-FEH activities were found for DH 307 and DH 338. FEH activities showed differential dynamics, depending on whether these activities were expressed on a fresh weight or on a protein level (Table S2), making results difficult to interpret. Based on specific activities on single samples, the 6-FEH activity in the lower parts may be proposed as a genotypic differentiation factor in fructan degradation between DH 307 and DH 338, but this requires further statistical analyses during the following growing seasons. Previous research had already pointed out that the lower parts of the stem had the highest WSC remobilization efficiency of dry matter toward the grain (Van Riet et al., 2006; Ehdaie et al., 2006b).

Overall time-dependent protein degradation may differ among different DH lines, and perhaps there may exist mechanisms to protect certain FEHs against protease-mediated degradation in senescing stem tissues. Moreover, stresses may result in vesicle-mediated exocytosis, carrying fructans to the apoplast (Valluru et al., 2008), where they may be subject of degradation by cell wall bound FEHs (Van Riet et al., 2006), which were not considered in our experiments focusing on soluble enzymes.

Since 2,6 linkages predominate in wheat stems, it is evident that some enzymes that can degrade this type of linkages (6-FEHs, 6&1-FEHs, 6-KEHs, or promiscuous INVs) are involved. The lack of commercially available 6-kestose and bifurcose as substrates complicates 6&1-FEH and 6-KEH activity measurements. Although it would be interesting to follow up the expression of 6&1-FEH and 6-KEH genes in senescing wheat stems, it can be speculated that FEHs may be regulated by specific FEH inhibitors *in planta*, further complicating the situation.

Sucrose transport from the lower parts to the developing grain includes the establishment of a sucrose gradient between “source” and “sink” tissues. Interestingly, no significant differences in sucrose content were found between treatments and segments among the two DH lines before 40 DAA, suggesting that genotype-specific sucrose gradients along the stem segments is not a likely determining factor of drought adaptation. Our data suggest that efficient fructan degradation, sucrose synthesis, and sucrose loading in the lower parts are essential aspects of drought adaptation. Therefore, a significant reduction of fructans in the lower parts of the stem between 20 and 30 DAA could be a diagnostic phenotype for rapid fructan remobilization.

Although the patterns of stem WSC were overall similar to those of fructans, our results indicate that stem WSC levels parallel those of fructan only during the period between 20 and 30 DAA. During this time, in irrigated plants, 70–80% of WSC were made up of fructan. Before and after this period the fructan proportion varied from 10 to 60% of WSC in a stem segment-dependent fashion. Under drought stress, the proportion of fructan varied depending on genotype and plant growth stage. Under drought, the degradation of fructan was more prominent than the reduction of total stem WSC levels.

From this, it can be concluded that the use of WSC as a drought tolerance marker is certainly not precise, mainly because of its dependency on growth stage and stress level. High stem WSC remobilization efficiency depends on fast fructan degradation, especially in the lower parts of the stem. It depends also on sucrose resynthesis and its subsequent transport to the grain. Therefore, fructan dynamics and FEH activities in the lower parts of the stem may be more useful indicators of remobilization efficiency.

This research highlights the importance of spatiotemporal studies of enzyme and metabolite levels on an array of different genotypes, resulting in a more functional picture than that provided by gene expression studies at whole-tissue level. Gene expression analysis fails to reflect the dynamics of enzymes due to tissue-specific post-transcriptional, translational and degradative regulatory mechanisms, illustrated by our own analysis of stem segments in this paper. Therefore, this work, by looking at the spatiotemporal distribution of specific degradative enzyme activities and metabolites, contributes toward an improved understanding of the mechanisms and dynamics of fructan oligosaccharide storage and remobilization and their roles in the grain-filling process in wheat.

### Conflict of interest statement

The authors declare that the research was conducted in the absence of any commercial or financial relationships that could be construed as a potential conflict of interest.

## Abbreviations

DH: double haploid
GW: grain weight per spike
KN: kernel number per spike
SSR: microsatellite molecular markers
TGW: thousand grain weight
WSC: water soluble carbohydrate.
