# Supplementary material for: Wheat genotypic variation in dynamic fluxes of WSC components in different stem segments under drought during grain filling
Source: Front Plant Sci. 2015 Aug 11;6:624. doi: 10.3389/fpls.2015.00624 (PMC4531436; doi:10.3389/fpls.2015.00624)
Supplement: Supplementary file 1 [file Table1.PDF]

**Supplementary Table 1.** Plant growth stages presented as days after anthesis at each sampling date

| DH lines | Anthesis dates (d/mm) | Sampling dates in 2011 (d/mm) | | | | | | |
|---|---|---|---|---|---|---|---|---|
| | | 30/08 | 6/09 | 14/09 | 20/09 | 28/09 | 4/10 | 14/10 |
| DH307 | 31/08 | -1 | 6 | 14 | 20 | 28 | 34 | 44 |
| DH 338 | 8/09 | -9 | -2 | 6 | 12 | 20 | 26 | 36 |
