# Supplementary material for: Wheat genotypic variation in dynamic fluxes of WSC components in different stem segments under drought during grain filling
Source: Front Plant Sci. 2015 Aug 11;6:624. doi: 10.3389/fpls.2015.00624 (PMC4531436; doi:10.3389/fpls.2015.00624)
Supplement: Supplementary file 2 [file Table2.PDF]

Supplementary Table 2. Comparison of 1-FEH and 6-FEH enzyme activities at fresh weight and protein level in different stem segments in DH 307 and DH 338 at 20, 26 and 28 DAA under drought and irrigated conditions in the field

DH lines and treatments		Protein/FW (mg/g)		1-FEH activity				6-FEH activity			
				By FW*		By protein		By FW		By protein	
Days after anthesis		20	28	20	28	20	28	20	28	20	28
DH 307 Irrigated	Peduncle	0.350	0.275	387	528	1107	1919	31	46	89	167
	Penultimate	0.050	0.058	373	347	7459	5957	32	31	634	539
	**Lower parts**	**0.025**	**0.025**	**336**	**304**	**13427**	**12143**	**27**	**31**	**1091**	**1233**
	Sheath	0.800	0.525	692	778	864	1482	74	101	93	193
DH 307 Drought	Peduncle	0.550	0.200	575	552	1046	2760	51	47	92	235
	Penultimate	0.025	0.042	353	438	14108	10506	36	42	1438	999
	**Lower parts**	**0.025**	**0.008**	**258**	**265**	**10328**	**31839**	**32**	**41**	**1296**	**4943**
	Sheath	0.800	0.325	698	809	873	2490	76	119	95	368
Days after anthesis		20	26	20	26	20	26	20	26	20	26
DH 338 Irrigated	Peduncle	0.175	0.163	467	585	2668	3597	42	53	238	324
	Penultimate	0.083	0.042	405	349	4855	8368	33	33	402	784
	**Lower parts**	**0.075**	**0.025**	**290**	**253**	**3870**	**10112**	**28**	**37**	**369**	**1478**
	Sheath	0.400	0.300	559	614	1398	2047	76	84	191	279
DH 338 Drought	Peduncle	0.313	0.150	770	627	2463	4182	66	67	211	449
	Penultimate	0.100	0.050	574	487	5736	9736	45	64	453	1278
	**Lower parts**	**0.033**	**0.025**	**456**	**430**	**13670**	**17202**	**38**	**67**	**1150**	**2673**
	Sheath	0.375	0.338	723	614	1928	1819	90	88	241	260

*By FW: nmol fructose min^{-1} g^{-1} FW (fresh weight); by protein: nmol fructose min^{-1} mg^{-1} protein
