# Supplementary material for: Wheat genotypic variation in dynamic fluxes of WSC components in different stem segments under drought during grain filling
Source: Front Plant Sci. 2015 Aug 11;6:624. doi: 10.3389/fpls.2015.00624 (PMC4531436; doi:10.3389/fpls.2015.00624)
Supplement: Supplementary file 4 [file Image2.PDF]

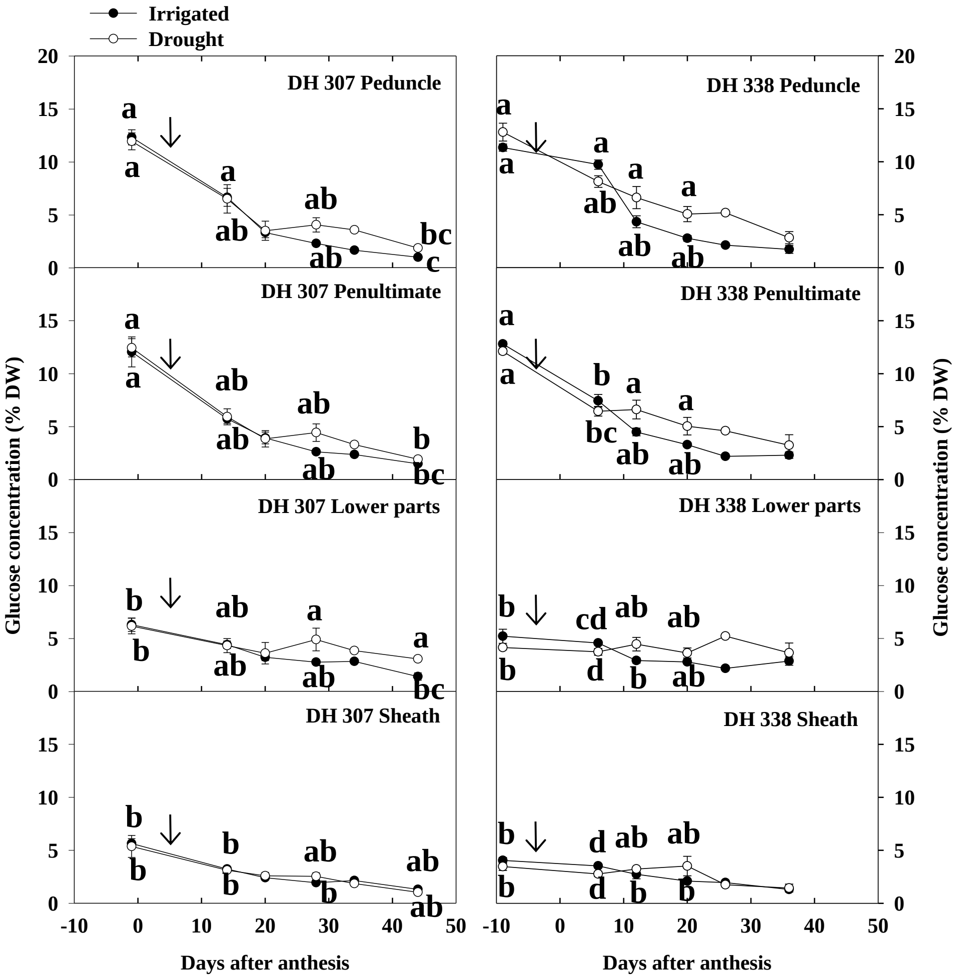

Supplementary Figure S2. Glucose concentration in different segments in DH 307 and DH 338 under drought (open circles) and irrigated conditions (closed circles) in the field. The vertical bars represent SE. Values with the same letter are statistically not different at $P = 0.05$. Arrows indicate start of drought treatment
