# Supplementary material for: Wheat genotypic variation in dynamic fluxes of WSC components in different stem segments under drought during grain filling
Source: Front Plant Sci. 2015 Aug 11;6:624. doi: 10.3389/fpls.2015.00624 (PMC4531436; doi:10.3389/fpls.2015.00624)
Supplement: Supplementary file 7 [file Image5.PDF]

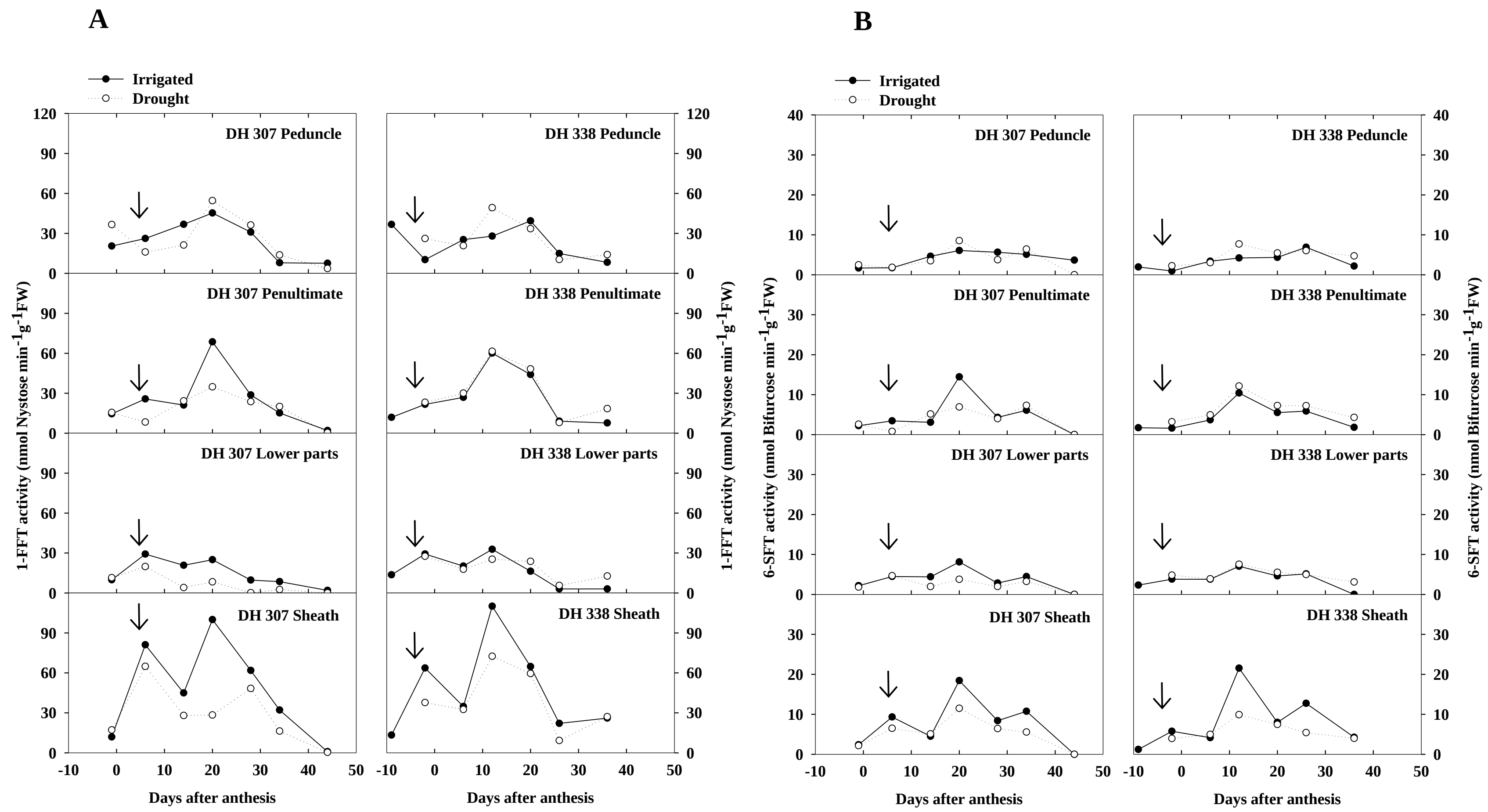

Supplementary Figure S5. Enzyme activities of 1-FFT **(A)** and 6-SFT **(B)** at fresh level in different segments in DH 307 and DH 338 under drought (open circles) and irrigated conditions (closed circles) in the field. Arrows indicate start of drought treatment
